# Early drop in systolic blood pressure, heart rate at admission, and their effects on worsening renal function in elderly patients with acute heart failure

**DOI:** 10.1186/s12872-020-01656-1

**Published:** 2020-08-10

**Authors:** Makoto Takeuchi, Michiaki Nagai, Keigo Dote, Masaya Kato, Noboru Oda, Eiji Kunita, Eisuke Kagawa, Aya Yamane, Yusuke Kobayashi, Haruko Shiota, Ayano Osawa, Hiroshi Kobatake

**Affiliations:** Department of Cardiology, Hiroshima City Asa Hospital, 2-1-1 Kabeminami, Aaskita-ku, Hiroshima, 731-0293 Japan

**Keywords:** Early drop in systolic blood pressure, Heart rate, Worsening renal function, Acute heart failure, Elderly

## Abstract

**Background:**

Regardless of patients’ baseline renal function, worsening renal function (WRF) during hospitalization is associated with poor outcomes. In individuals with acute heart failure (AHF), one predictor of WRF is an early drop in systolic blood pressure (SBP). Few studies have investigated WRF in elderly AHF patients or the influence of these patients’ heart rate (HR) at admission on the relationship between an early SBP drop SBP and the AHF.

**Methods:**

We measured the SBP and HR of 245 elderly AHF inpatients (83 ± 6.0 years old, females 51%) at admission and another six times over the next 48 h. We defined ‘WRF’ as a serum creatinine increase ≥0.3 mg/dL by Day 5 post-admission. We calculated the ‘early SBP drop’ as the difference between the admission SBP value and the lowest value during the first 48 h of hospitalization.

**Results:**

There were significant differences between the 36 patients with WRF and the 209 patients without WRF: early SBP drop (51 vs. 33 mmHg, *p* < 0.01) and HR at admission (79 vs. 90 bpm, *p* < 0.05), respectively. In the multiple logistic regression analysis adjusted for the confounders, higher early SBP drop (*p* < 0.04) and lower HR at admission (*p* < 0.01) were significantly associated with WRF. No significant association was shown for the interaction term of early SBP drop × HR at admission with WRF.

**Conclusions:**

In these elderly AHF patients, exaggerated early SBP drop and lower HR at admission were significant independent predictors of WRF, and these factors were additively associated with WRF.

## Background

Approximately one-third of individuals with acute heart failure (AHF) experience worsening renal function (WRF) during hospitalization [1–3], and it has been demonstrated that WRF has a strong association with poorer patient outcomes regardless of the patients’ baseline renal function [1–5]. It is thus crucial to identify patients who are at risk of developing WRF, at the earliest point possible. Higher blood pressure (BP) at a patient’s admission to a hospital was shown to be linked to a greater risk of WRF [3, 6, 7], and a drop in a patient’s systolic blood pressure (SBP) during the first days post-admission has also been shown to pose a risk of the development of WRF [1, 8, 9] and to be associated with the prognosis [9, 10]. It was suggested that AHF patients’ baseline heart rate (HR) can be used to predict in-hospital cardiac mortality, and in contrast to patients with chronic heart failure (CHF), among AHF patients a lower HR at baseline was shown to be associated with a higher in-hospital rate of cardiac death [4].

Few investigations have examined the relationships among WRF, early SBP drop, and the HR at admission in elderly patients with AHF. Herein, we tested our hypothesis that an early drop in the SBP of an elderly AHF patient could be used to predict WRF in the patient. We also investigated the effect of HR at admission on any interactions among these factors.

## Methods

### Study population

This prospective observational cohort study was conducted at Hiroshima City Asa Hospital from January 2013 to December 2015. We considered a patient as eligible for study enrollment if he or she were hospitalized for AHF during this study. AHF was defined as a rapid onset or worsening of symptoms and/or signs of heart failure (HF). The HF symptoms were: fatigue, breathlessness, and ankle swelling. The signs were: peripheral edema, elevated jugular venous pressure, and pulmonary crackles [11, 12]. Each patient’s diagnosis of AHF was based on the 2016 guidelines of the European Society of Cardiology for the diagnosis of heart failure [12].

Regarding the B-type natriuretic peptide (BNP) used for the diagnosis of AHF, we set an exclusion cut-off point at 100 pg/mL based on current guidelines [12]. Each AHF diagnosis was made by experienced cardiologists. We excluded patients with multiple organ failure, shock, or sepsis, and those who were on chronic hemodialysis. The consecutive eligible elderly patients with AHF over 70 years of age were enrolled after the purpose of study was fully explained to the patients.

### Clinical scenario

The clinical scenario is based on initial SBP on admission and other symptoms, and is one of the most commonly used clinical classifications [13]. In summary, AHF patients were divided into five groups: clinical scenario 1 (SBP > 140 mmHg with dyspnea and/ or congestion), clinical scenario 2 (SBP 100–140 mmHg with dyspnea and/or congestion), clinical scenario 3 (SBP < 100 mmHg with dyspnea and/or congestion), clinical scenario 4 (signs of acute coronary syndrome with dyspnea and/or congestion), and clinical scenario 5 (isolated right ventricular failure) [13]. This classification is useful in determining initial treatment (e.g., non-invasive positive pressure ventilation, vasodilators, inotropes, and diuretics). This urgent/immediate approach shortens the length of hospital stay and reduces in-hospital mortality in terms of shortening the time to the start treatment [14, 15].

### Procedures

The physician investigators were not prohibited from using any standard medication thought necessary to treat the enrolled patients, including additional vasodilators [1]. Participants’ BP and HR were measured at admission, and six more recordings were performed within 48 h of admission. At every recording, BP and HR were measured 3 times, and the mean values of the second and third readings at each recording were used. At the baseline and 12 h later, serum creatinine was measured. After that point, serum creatinine was measured daily through Day 5 post-admission [1].

### Definitions

We defined ‘WRF’ as a ≥ 0.3 mg/dL increase in the patient’s serum creatinine level compared to his or her baseline value, at any time through Day 5 [1]. We examined the potential ‘early drop in SBP’ as the difference between the patient’s SBP value taken at admission and the lowest SBP value measured during the first 48 h of hospitalization [1]. For all of the BP measurements, the patient was supine and had rested for ≥5 min.

### Statistical analyses

The data are presented as the mean ± the standard deviation (SD) or as a percentage, and all analyses were performed with SPSS ver. 11.5 J software (SPSS, Chicago, IL). We used the Chi-squared test to compare categorical variables among groups, and we performed one-way analysis of variance (ANOVA) for the continuous variables to tested the null hypothesis that the means of early SBP drop and HR at admission were same between the group with WRF and that without WRF. To estimate and test the independent effects of early SBP drop and the HR at admission on WRF, we conducted a multiple logistic regression analysis. In addition to the patient age, gender and other possible confounders, those factors that would contribute to the outcome in the initial univariable analyses at *p* values of less than 0.1 were considered as candidate variables for the multivariable model. As the distribution of BNP was highly skewed, log transformation was carried out. Probability (p)-values < 0.05 were accepted as significant.

## Results

### Patient characteristics

**Fig. 1:**
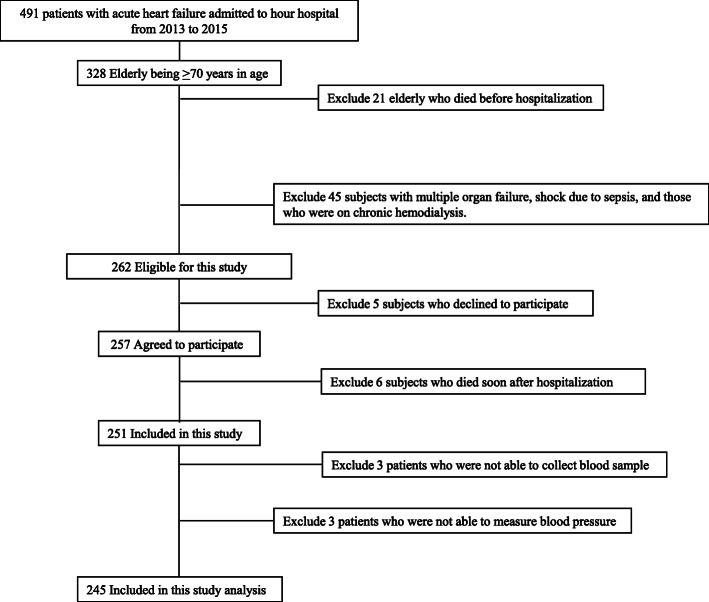
Study sample inclusion and exclusion flow chart

The 491 patients with AHF admitted to hour hospital from January 2013 to December 2015, and we included 251 elderly patients with AHF in this study. While 3 patients who were not able to collect blood sample were excluded, 3 patients who were not able to measure blood pressure were excluded. Ultimately, we analyzed 245 patients in this study. Figure 1 summarizes the flow of potential participants.

Creatinine values that allowed a classification of WRF were available for all 245 of the enrolled patients: mean age 82.9 ± 6.0 years, 124 females (50.6%), 121 males (49.4%). This study population was consisted from a mix of 166 patients with newly arisen (“de novo”) AHF (66%) and 79 patients with acutely decompensated chronic heart failure (34%). Among all patients, 78.3% had a history of hypertension, and 11.8% had ischemic heart failure. There were patients with the 3 patients with sinus bradycardia (1.2%), 12 patients with sinoatrial block (4.9%) and 8 patients with atrio-ventricular block (3.3%). According to with and without WRF, there were no significant difference in sinus bradycardia (*p* = 0.4), sinoatrial block (*p* = 0.5) or atrio-ventricular block (*p* = 0.2).

### Prevalence and clinical correlates of WRF

WRF was confirmed in 14.7% (*n* = 36) of the 245 evaluable patients. Table 1 summarizes the characteristics of patients with and without WRF. Compared to the 209 patients without WRF (85.3%), those with WRF had significantly higher prevalences of a history of hypertension, intravenous loop diuretic use, and intravenous nitroglycerin use; in addition, the WRF group had significantly higher baseline SBP values, significantly lower HR at admission, and significantly greater early drops in SBP through the first 48 h post-admission (Table 1).

**Table 1:** Baseline characteristics of the elderly acute heart failure patients with and without worsening renal function

Baseline characteristics	Total (***n*** = 245)		
		WRF	
		Yes (n = 36)	No (***n*** = 209)
Age, yrs	82.9 ± 6.0	82.3 ± 6.4	83.1 ± 6.0
Male, %	49.4	41.7	50.7
Body weight at assessment, kg	50.1 ± 12.0	48.4 ± 14.3	50.5 ± 11.5
Clinical scenario 1, %	41.2	58.3*	38.3
Clinical scenario 2, %	30.6	16.7*	33.0
Clinical scenario 3, %	8.2	8.3	8.1
Clinical scenario 4, %	13.1	13.9	12.4
Ejection fraction, %	45.6 ± 14.7	42.5 ± 13.0	46.2 ± 14.9
Current smoking, %	25.3	27.8	24.9
Daily alcohol intake, %	20.4	11.1	22.0
Hypertension, %	78.3	91.7*	76.0
Diabetes mellitus, %	33.9	33.3	34.0
Lipid disorder, %	44.4	44.4	38.8
Chronic atrial fibrillation, %	23.6	22.2	23.9
Baseline laboratory values:			
Log BNP	6.07 ± 1.2	6.38 ± 0.9	6.01 ± 1.2
Hemoglobin, g/dL	11.8 ± 2.1	11.7 ± 2.2	11.8 ± 2.1
BUN, mg/dL	31.5 ± 17.3	30.7 ± 16.5	31.6 ± 17.5
Serum creatinine, g/dL	1.50 ± 1.0	1.61 ± 1.0	1.47 ± 1.0
Medication at baseline:			
ACEI use, %	8.17	2.78	9.1
ARB use, %	45.3	52.8	44.0
CCB use, %	40.0	36.1	40.7
Beta-blocker, %	29.0	36.1	27.8
Loop diuretics, %	50.0	58.3	48.6
Medication during admission:			
I.V. loop diuretic use, %	75.5	88.9*	73.2
I.V. isosorbide dinitrate, %	27.8	50.0**	23.9
I.V. carperitide use, %	7.8	5.6	8.1
Blood pressure and heart rate parameters:			
Systolic blood pressure admission, mmHg	140 ± 32	158 ± 37***	137 ± 30
Diastolic blood pressure at admission, mmHg	76.5 ± 19	78.5 ± 18	76.1 ± 20
Heart rate at admission, mmHg	88.1 ± 28	79.3 ± 21*	89.6 ± 29
Early systolic blood pressure drop, mmHg	35.3 ± 31	51.3 ± 30***	32.5 ± 30

Data are mean ± standard deviation or as percentages. Analysis of variance for continuous variables and the χ^2^ test for qualitative variables were used.*p < 0.05, **p < 0.01, ***p < 0.001 compared to the no-worsening renal function group. BNP indicates B-type natriuretic peptide; BUN, blood urea nitrogen; ACEI, angiotensin-converting enzyme inhibitor; ARB, angiotensin II receptor blocker; CCB, calcium channel blocker; I.V., intravenous; WRF, worsening renal function

### Factors associated with worsening renal function on univariate analysis

As shown in Table 2, in the univariate logistic regression analysis, the following were significantly associated with WRF: hypertension (odds ratio [OR] 3.48; 95% confidence interval [CI] 1.02–11.8), intravenous nitroglycerin (OR 3.18; 95%CI 1.54–6.58), SBP at admission (OR 1.02; 95%CI 1.01–1.03), HR at admission (OR 0.987; 95%CI 0.97–0.999), and early SBP drop (OR 1.02; 95%CI 1.01–1.03).

**Table 2:** Univariate logistic regression analysis between early systolic blood pressure drop and worsening renal function

Baseline characteristics	Odds ratio	95% CI	*p*-value
Age, yrs	0.98	0.92–1.04	0.5
Male, yes = 1, no = 0	0.69	0.34–1.42	0.3
Body weight at assessment, kg	0.99	0.96–1.02	0.4
Clinical scenario 1, yes = 1, no = 0	1.42	0.64–3.18	0.4
Clinical scenario 2, yes = 1, no = 0	1 (Ref.)	–	–
Clinical scenario 3, yes = 1, no = 0	1.04	0.27–3.99	0.96
Clinical scenario 4, yes = 1, no = 0	1.33	0.43–4.08	0.6
Ejection fraction, %	0.18	0.96–1.01	0.2
Current smoking, yes = 1, no = 0	1.16	0.53–2.57	0.7
Daily alcohol intake, yes = 1, no = 0	0.14	0.15–1.32	0.1
Hypertension, yes = 1, no = 0	3.48	1.02–11.8	0.046
Diabetes mellitus, yes = 1, no = 0	0.97	0.46–2.06	0.9
Lipid disorder, yes = 1, no = 0	1.23	0.62–2.58	0.5
Chronic atrial fibrillation, yes = 1, no = 0	0.91	0.39–2.12	0.8
Baseline laboratory values:			
Log BNP	1.36	0.97–1.91	0.08
Hemoglobin, g/dL	0.97	0.82–1.15	0.7
BUN, mg/dL	0.997	0.98–1.02	0.8
Serum creatinine, g/dL	1.12	0.83–1.53	0.5
Medication at baseline:			
ACEI use, yes = 1, no = 0	0.29	0.04–2.20	0.2
ARB use, yes = 1, no = 0	1.42	0.70–2.89	0.3
CCB use, yes = 1, no = 0	0.83	0.40–1.72	0.6
Beta-blocker, yes = 1, no = 0	1.47	0.70–3.01	0.3
Loop diuretics, yes = 1, no = 0	1.48	0.73–3.04	0.3
Medication during admission			
I.V. loop diuretic use, yes = 1, no = 0	2.93	0.99–8.65	0.052
I.V. isosorbide dinitrate, yes = 1, no = 0	3.18	1.54–6.58	0.002
I.V. carperitide use, yes = 1, no = 0	0.66	0.15–3.01	0.6
Blood pressure and heart rate parameters			
Systolic blood pressure at admission, mmHg	1.02	1.01–1.03	0.001
Diastolic blood pressure at admission, mmHg	1.01	0.99–1.03	0.5
Heart rate at admission, mmHg	0.987	0.97–0.999	0.046
Early systolic blood pressure drop, mmHg	1.02	1.01–1.03	0.001

Each parameters was included to the simple logistic regression model for worsening renal function. Patients with clinical scenario 2 were the reference (Ref.) group. BNP indicates B-type natriuretic peptide; BUN, blood urea nitrogen; ACEI, angiotensin-converting enzyme inhibitor; ARB, angiotensin II receptor blocker; CCB, calcium channel blocker; I.V., intravenous

### Determinants of WRF

The results of our multiple logistic regression analysis are presented in Table 3. The analysis was adjusted for the following confounders: patient age and gender, the left ventricular ejection fraction, the presence/absence of hypertension, beta-blocker use at baseline and log BNP, intravenous loop diuretic, isosorbide dinitrate and carperitide use. The two factors that were significantly associated with WRF were the patient’s HR at admission (adjusted OR [AOR] 0.98; 95%CI 0.96–0.99) and early SBP drop (AOR 1.02; 95%CI 1.004–1.03) (Table 3). The interaction term of early SBP drop × HR at admission was not significantly associated with WRF (*p* = 0.3).

**Table 3:** Logistic regression model of early systolic blood pressure drop for subsequential worsening renal function

Trait	Adjusted odds ratio	95% CI	***p***-value
Age, yrs	0.95	0.88–1.02	0.13
Male, yes = 1, no = 0	0.54	0.22–1.33	0.18
Ejection fraction, %	0.97	0.94–1.01	0.09
Hypertension, yes = 1, no = 0	2.93	0.77–11.1	0.12
Beta-blocker at baseline, yes = 1, no = 0	1.44	0.60–3.44	0.41
Log BNP	1.05	0.65–1.70	0.85
I.V. loop diuretic use during admission, yes = 1, no = 0	2.21	0.58–8.35	0.24
I.V. isosorbide dinitrate during admission, yes = 1, no = 0	1.80	0.73–4.48	0.20
I.V. carperitide use during admission, yes = 1, no = 0	0.68	0.13–3.57	0.65
Heart rate at admission, bpm	0.98	0.96–0.99	0.004
Early systolic blood pressure drop, mmHg	1.02	1.004–1.03	0.02

Parameter were added simultaneously to the regression model. BNP indicates B-type natriuretic peptide; I.V., intravenous

## Discussion

The results of this observational study demonstrated that a greater drop in SBP within the first 48 h after the hospitalization of an elderly patient with AHF — as well as the patient’s HR at admission — were independently associated with a higher risk of the occurrence of WRF. This is the first investigation to report associations of an early SBP drop and the HR at admission with WRF in patients with AHF.

### Early SBP drop and WRF

Our findings revealed that an early SBP drop after hospitalization in elderly patients with AHF was an independent determinant of the development of WRF. In the univariate analyses conducted in prior studies, the baseline SBP level itself was found to be positively correlated with WRF, and in those studies [6, 7], a higher risk of WRF during hospitalization was observed in AHF patients with hypertension. Forman et al. [3] reported that an SBP value at admission > 160 mmHg was independently associated with an increased WRF risk. In the present study, although the baseline SBP level was significantly positively correlated with the early SBP drop (r = 0.82, *p* < 0.001), not baseline SBP level (OR 1.015; 95%CI 0.999–1.03; *p* = 0.062) but early SBP drop remained independently related to a higher risk of WRF when either the baseline SBP level or the early SBP drop was included in the multiple regression model. This result suggests that a larger drop in SBP (and not a higher baseline SBP) was a significant risk factor for WRF in this series of elderly AHF patients.

We suspect that our present findings might be due to the auto-regulatory response in the kidneys [16]. The kidneys’ vascular system will constrict (its afferent arterioles, specifically) when the renal perfusion pressure rises because of an increase in BP; the kidneys’ inter-lobal arteries may also constrict. Afferent vasodilation occurs when the BP drops, and if the BP falls further, efferent vasoconstriction can also occur. The kidneys are thus able to maintain — over a wide range of BP values — constant glomerular capillary perfusion, pressure, and filtration. The pre-glomerular circulation of patients with long-standing hypertension has shown a blunted ability to dilate in response to a drop in SBP, and this can result in an exaggerated decrease in the intraglomerular pressure [17]. Our present observation that a higher drop in SBP is related to a higher risk of WRF is consistent with the above explanation, but our findings remain to be confirmed in further studies.

Although our results indicate that the use of an intravenous loop diuretic or isosorbide dinitrate during a patient’s hospital admission might be positively associated with WRF, neither loop diuretic nor isosorbide dinitrate use was shown to be a significant indicator of WRF in the multivariate model. Diuretics are known to present a risk of impaired kidney function in patients with heart failure, probably by a so-called ‘tubuloglomerular feedback’ mechanism. The distal tubules of the kidneys sense a loss of salt, and this leads to a release of adenosine, which then binds to the adenosine A1-receptor. Afferent vasoconstriction is the result, with a subsequent reduction in the renal blood flow (a main determinant of renal function in heart failure patients) [18].

Nitrate is thought to dilate the renal microvascular flow and increase the renal blood flow when other aspects of the vascular status are normal. However, a systemic vasodilation of the systemic vasculature might completely decrease the renal blood flow in accord with an acute SBP reduction. Each deleterious impact of diuretics or nitrate on the renal blood flow would thus be observed in an AHF population such as our present patients, because both diuretics and nitrate would contribute to a higher drop in SBP during the acute phase of HF.

### HR at admission and WRF

Our analyses revealed that the at-admission HR values of the elderly patients with AHF were independently associated with a higher risk of WRF. This finding confirms that a lower HR at admission may be a marker and may also pose a direct increased risk of WRF in AHF patients [19]. In individuals with chronic HF, elevated resting HR was reported to be associated with increased risks of cardiovascular disease and mortality [20, 21]. In the hyperacute phase of AHF, tachycardia is a mostly beneficial physiological compensatory response. An increase in the HR is necessary to maintain the cardiac output, due to structural limitations of the stroke volume [22].

Although the details of the relationship between the pathophysiology of AHF and the HR remain unknown [23], our present data demonstrate that a lower HR at baseline in patients with AHF was associated with a much higher risk of WRF. Bainbridge showed in 1915 that rapid volume loading results in increases of both blood pressure and heart rate [24], and a 2009 review showed that a higher baseline SBP is associated with a better outcome [25]. We speculate that in the urgent phase of AHF, a higher risk of WRF might be associated with an impaired ability to increase the heart rate to appropriate levels.

It should be noted that we did not observe a significant association between WRF and the interaction term of early SBP drop × HR at admission. We thus suggest that the early SBP drop and HR at admission each have an additive impact on WRF rather than a synergistic effect.

### Study limitations

This study is subject to regression dilution bias due to measurement error associated with high intra-person variability in HR and SBP. This bias is likely to have led to an underestimation in the strength of the association between either HR or SBP and WRF. This study could not refute the possibility of significant interaction between early SBP drop and HR at admission because there might not be enough power to detect that interaction. There is possibility of residual confounding because every possible confounder could not be adjusted for.

## Conclusion

Among the 245 elderly patients hospitalized for AHF, WRF was independently predicted by a greater drop in SBP during the first 48 h of hospitalization. The patients’ HR at admission also independently predicted WRF. An early SBP drop and the HR at admission might serve as additive surrogate markers of clinical outcomes in AHF patients.

## Data Availability

The datasets generated during and/or analysed during the current study are not publicly available due to data privacy regulation by Hiroshima City Asa Hospital.

## Abbreviations

WRF: Worsening renal function
AHF: Acute heart failure
BP: Blood pressure
SBP: Systolic blood pressure
HR: Heart rate
CHF: Chronic heart failure
BNP: B-type natriuretic peptide
SD: Standard deviation
ANOVA: Analysis of variance
OR: Odds ratio
CI: Confidential interval
